# Dysfunction of metabolic activity of bone marrow mesenchymal stem cells in aged mice

**DOI:** 10.1111/cpr.13191

**Published:** 2022-01-27

**Authors:** Xiaoyu Li, Xue Wang, Chunmei Zhang, Jinsong Wang, Songlin Wang, Lei Hu

**Affiliations:** ^1^ 159331 Molecular Laboratory for Gene Therapy and Tooth Regeneration Beijing Key Laboratory of Tooth Regeneration and Function Reconstruction Capital Medical University School of Stomatology Beijing China; ^2^ Beijing Laboratory of Oral Health Capital Medical University School of Basic Medicine Beijing China; ^3^ Immunology Research Center for Oral and Systemic Health Beijing Friendship Hospital Capital Medical University Beijing China; ^4^ Laboratory for Oral and General Health Integration and Translation Beijing Tiantan Hospital Capital Medical University Beijing China; ^5^ 159331 Department of Prosthodontics Capital Medical University School of Stomatology Beijing China

**Keywords:** aging, bone marrow mesenchymal stem cell, metabolic activity, metabolism, mitochondrial, osteogenesis

## Abstract

**Objectives:**

Evidences have suggested that the metabolic function is the key regulator to the fate of MSCs, but its function in senescence of MSC and the underlying mechanism is unclear. Therefore, the purpose of this study was to investigate the metabolic activity of MSCs and its possible mechanism during aging.

**Materials and Methods:**

We used the Seahorse XF24 Analyzer to understand OCR and ECAR in BMSCs and used RT‐PCR to analyze the gene expression of mitochondrial biogenesis and key enzymes in glycolysis. We analyzed BMSC mitochondrial activity by MitoTracker Deep Red and JC‐1 staining, and detected NAD+/NADH ratio and ATP levels in BMSCs. Microarray and proteomic analyses were performed to detect differentially expressed genes and proteins in BMSCs. The impact of aging on BMSCs through mitochondrial electron transport chain (ETC) was evaluated by Rotenone and Coenzyme Q10.

**Results:**

Our results demonstrated that the oxidative phosphorylation and glycolytic activity of BMSCs in aged mice were significantly decreased when compared with young mice. BMSCs in aged mice had lower mitochondrial membrane potential, NAD+/NADH ratio, and ATP production than young mice. FABP4 may play a key role in BMSC senescence caused by fatty acid metabolism disorders.

**Conclusions:**

Taken together, our results indicated the dysfunction of the metabolic activity of BMSCs in aged mice, which would play the important role in the impaired biological properties. Therefore, the regulation of metabolic activity may be a potential therapeutic target for enhancing the regenerative functions of BMSCs.

## Graphical abstract

Our results provide new insights into the differences in metabolic characteristics of bone marrow mesenchymal stem cells between young and old mice, and found that FABP4 is related to the senescence and metabolic activity of bone marrow mesenchymal stem cells. These proteins may be the potential regulators regulating the metabolic activity of BMSC senescence.

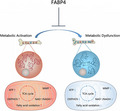

## 1 INTRODUCTION

Mesenchymal stem cells (MSCs) are pluripotent nonhematopoietic progenitor cells that have the capability of self‐renewal, multidirectional differentiation, and immunoregulation, and their pluripotency makes them an ideal source of regenerative cell therapy.^1^, ^2^, ^3^, ^4^ However, previous studies have shown that the functional properties of MSCs would be impaired due to the aging or disease status of donors, which may significantly affect the eventual efficiency.^5^, ^6^ Aging is a complex, gradual, and inevitable physiological process, accompanied by the accumulation of damage macromolecules, leading to organ dysfunction and the senescence of MSCs, with the character of significantly impaired biological properties of MSCs.^7^, ^8^, ^9^ Several methods have been tried to modify the senescence of MSCs, but the effect remains unsatisfied.^10^, ^11^

In recent years, more and more evidences show that the mitochondrial dynamics and function play the key function in the biological function of MSCs.^12^ Mitochondria are important organelles in eukaryotic cells, which is responsible for the production of cell energy and essential for maintaining tissue homeostasis. The mitochondrial energy metabolism supports cell survival and proliferation, and determines different cell states.^13^ The typical function of mitochondria as "cell dynamics" is to produce adenosine triphosphate (ATP) by oxidative phosphorylation (OXPHOS) and support anabolism by producing TCA cycle metabolites (such as citrate).^14^ In addition, MSCs rely on glycolysis to produce ATP are usually rich in fully functional mitochondria,^15^ which means that stem cells integrate different metabolites produced by glycolysis and mitochondrial metabolism to regulate their fate and function. Interestingly, several recent studies have shown that there are differences in mitochondrial metabolism among different cell types,^16^, ^17^ and the accumulation of mitochondrial DNA point mutations may lead to the dysfunction of different stem cells.^18^, ^19^ However, whether aging affects the metabolic activity of MSCs and the possible mechanism remains unclear.

Therefore, in this study, we aimed to investigate the metabolic activity of bone marrow–derived MSCs (BMSCs) and its possible mechanism in young and aged mice. Our data suggest that metabolic activity may be a potential therapeutic target for the regeneration of senescent BMSCs and the treatment of age‐related bone loss. In addition, these results provide an important theoretical basis for later cell therapy and tissue regeneration applications.

## 2 MATERIALS AND METHODS

### 2.1 Mice

This study was approved by the Animal Care and Use Committee of Capital Medical University. C57BL/6 mice, purchased from Beijing Vital River Laboratory Animal (Beijing, China), were randomly divided into two groups (*n* = 10/group), including the young group (2‐month‐old, 2 M) and the elderly group (18‐month‐old, 18 M). Mice were maintained in a specific pathogen‐free animal facility and kept under conventional conditions with free access to water and food.

### 2.2 Cell culture of BMSCs

Young (2 M) BMSCs and elderly (18 M) BMSCs were cultured from bone cavity of femurs and tibias of 2‐month‐aged and 18‐month‐aged mice, respectively. The cells were cultured with alpha‐MEM medium (Gibco) supplemented with 20% fetal bovine serum (FBS, Gibco), 2‐mM glutamine, 100‐U/mL penicillin, and 100‐mg/mL streptomycin (Invitrogen), and then incubated in 5% carbon dioxide at 37°C. BMSCs between passages 3–4 were used in the following study. In this study, 50‐nM Rotenone (Rot, Sigma‐Aldrich) and 30‐μM Coenzyme Q10 (CoQ10, Sigma‐Aldrich) were used to stimulate the BMSCs.

### 2.3 Flow cytometric analysis

The cell surface markers of 2 M BMSCs and 18 M BMSCs were analyzed by flow cytometry (BD Biosciences) according to the manufacturer's instructions. Briefly, after washing with PBS containing 3% FBS, the cells were incubated with monoclonal antibodies against mouse CD105, SCA1, CD90, CD31, CD45, and CD11b in the dark at 4°C for 1 h; cells without pretreatment by any antibody were used as blank control. Subsequently, the cells were washed with PBS and analyzed by flow cytometry.

To detect the proliferation of 2 M BMSCs and 18 M BMSCs in vivo, bone marrow cells from bone cavity of femurs and tibias of mice were washed out with staining buffer (PBS with 1% FBS, 0.09% NaN_3_) and treated with erythrocyte lysate (Solarbio) for 3 min, then resuspended to a concentration of 2 × 10^6^/ml. The cells were incubated with monoclonal antibodies against mouse CD105, SCA1, CD90, CD45, and CD11b in the dark at 4 °C for 1 h and washed with staining buffer. After using Foxp3/transcription factor staining buffer (eBioscience) to break the cell nuclear membrane, the cells were incubated with a monoclonal antibody against mouse Ki67 in the dark at 4 °C for 1 h. Subsequently, the cells were washed with staining buffer and analyzed by flow cytometry.

### 2.4 CFSE labeling for cell proliferation analysis

Single‐cell suspensions (1 × 10^6^ cells) from 2 M BMSCs and 18 M BMSCs were separately incubated with 5‐mM carboxyfluorescein succinimidyl ester (CFSE) for 10 min in prewarmed 0.1% bovine serum albumin (BSA). Five volumes of ice‐cold medium were added to stop the staining process, and cells were washed three times with the culture medium. The cells were then seeded onto 6‐well plates (BD Biosciences) at 2 × 10^5^ cells/well; after 3 days cultured, they were trypsinized. Flow cytometry was used to analyze cell proliferation index. The results were represented as mean ± standard deviation of three independent experiments performed in triplicate.

### 2.5 Multilineage differentiation potential detection

To evaluate the osteogenic differentiation potential, 1 × 10^5^ 2 M BMSCs and 18 M BMSCs were separately seeded onto 6‐well plates (BD Biosciences) and cultured with osteogenic inductive medium (Cyagen). After 7 days, the BMSCs were stained with ALP. The level of ALP activity in the cells was measured using an ALP activity assay kit (Nanjing Jiancheng Bioengineering Institute) according to the manufacturer's instructions, and the absorbance was measured by a microplate reader at a wavelength of 520 nm. After 21 days, the mineralized nodules were stained with 2% Alizarin Red. The mineralization was measured by using the Image‐Pro Plus 6.0 program (Media Cybernetics).

To evaluate the adipogenic differentiation potential, 1 × 10^5^ 2 M BMSCs and 18 M BMSCs were separately seeded onto 6‐well plates (BD Biosciences) and cultured with adipogenic inductive medium (Cyagen). After 14 days, the lipid droplets were stained by Oil Red O. The area of the Oil Red O stain was measured by a microplate reader at a wavelength of 510 nm after solubilizing in isopropanol (Sigma‐Aldrich) for 30 min at the room temperature.

To evaluate the chondrogenic differentiation potential, 1 ×10^5^ 2 M BMSCs and 18 M BMSCs were separately seeded onto 6‐well plates (BD Biosciences) and cultured with chondrogenic inductive medium (Cyagen). After 21 days, the synthesis of proteoglycans by chondrocytes was stained by Alcian Blue. The area of the Alcian Blue stain was measured by a microplate reader at a wavelength of 600 nm after solubilizing in guanidine hydrochloride solution (Sigma‐Aldrich) overnight at 4°C.

### 2.6 Angiogenic analysis

Tube formation assays were performed using Matrigel Basement Membrane Matrix (BD Biosciences) according to the manufacturer's instructions to assess angiogenesis in vitro. Briefly, Matrigel (50 μl/well) was thawed overnight at 4°C and added to a 96‐well plate using a cold pipette tip. The plates were then incubated at 37°C until Matrigel solidified. Next, 2 M BMSCs and 18 M BMSCs (4 × 10^4^ cells/well) were seeded separately in complete medium. After incubation at 37°C for 4 h and 8 h, tube formation was assessed with an inverted microscope. The total tubule length, Nb nodes, and Nb meshes were measured using ImageJ software (National Institutes of Health).

### 2.7 Oxygen consumption rate (OCR) and extracellular acidification rate (ECAR) analysis

OCR and ECAR were measured using a seahorse XF24 analyzer (Seahorse bioscience). Briefly, seeding 2 M BMSCs and 18 M BMSCs (4 × 10^4^ cells per well) in XFe24 Cell Culture Microplates. For OCR measurement, cells were covered with 500‐µL assay medium (XF base medium (Seahorse, 102353), 1‐mM sodium pyruvate, 1‐mM L‐glutamine, and 10‐mM glucose). Port injections were performed with 1‐mM oligomycin, 3‐mM FCCP, 0.5‐mM antimycin, and rotenone. For ECAR measurement, cells were covered with 500‐µl assay medium (XF base medium, 1‐mM L‐glutamine). Port injections were performed with 10‐mM glucose, 1‐mM oligomycin, and 50‐mM 2‐DG.

### 2.8 Mitochondrial activity

2 M BMSCs and 18 M BMSCs (1 × 10^5^ cells/ml) were incubated in alpha‐MEM medium (Gibco) at 37°C with either (a) MitoTracker Deep Red (Invitrogen) 200 nM for 30 mins, (b) JC‐1 (Sigma) 10 ug/mL for 30 mins, as per the manufacturer's instructions. Cells were then washed with PBS and stained for HSPC markers, as described above, and analyzed immediately.

### 2.9 NAD+/NADH quantification

Wash 2 M BMSCs and 18 M BMSCs with cold PBS. Pellet 2 × 10^5^ cells for each assay in a microcentrifuge tube by centrifuging at 250 *g* for 5 mins. Extract cells with 400 μl of NADH/NAD extraction buffer by homogenization or freeze/thawing for two cycles of 20 min on dry ice followed by 10 min at room temperature. Centrifuge the samples at 13,000 × *g* for 10 min to remove insoluble material. As the manufacturer's instructions, measure the absorbance at 450 nm (A450).

### 2.10 ATP determination

ATP was determined using ATP assay kit (Sigma). Briefly, ATP concentration is determined by phosphorylating glycerol, resulting in a colorimetric (570 nm) product proportional to the amount of ATP present. Separately lyse 2 M BMSCs and 18 M BMSCs (3 or 4 passages) (1 × 10^6^) in 100 uL of ATP assay buffer. As the manufacturer's instructions, measure the absorbance at 570 nm (A570) in a microplate reader.

### 2.11 RT‐PCR analysis

Total RNA was isolated from 2 M BMSCs and 18 M BMSCs using the RNAios Plus reagent (Takara) and was reverse transcribed to cDNA using the PrimeScript TM RT Reagent Kit with gDNA Eraser (Takara). The cDNAs were amplified by PCR with the following thermocycler conditions: 95 ℃ for 2 min, then 40 cycles of 95 ℃ for 15 s, and 60 ℃ for 1 min. *GAPDH* was used as an internal control to quantify and normalize the results. The information on the primers is shown in Table S1 (Additional file 1).

### 2.12 Western blot analysis

2 M BMSCs and 18 M BMSCs were washed three times with ice‐cold PBS and lysed using RIPA reagent containing 1% PMSF and 1% phosphatase inhibitor cocktail. After centrifugation at 14,000 *g* for 5 min, total protein concentrations were measured using a BCA Protein Assay Kit (Solarbio). Proteins were then separated by sodium dodecyl sulphate–polyacrylamide gel electrophoresis (SDS‐PAGE) according to molecular weight and transferred onto polyvinylidene difluoride (PVDF) membranes (Millipore). The membranes were blocked with 5% milk for 2 h and then incubated with primary antibodies overnight at 4°C. Next, the membranes were washed three times with Tris‐buffered saline solution with Tween‐20 (TBS‐T) and incubated with secondary antibodies at room temperature for 1 h. The protein bands were then developed with the use of the Pierce ECL Western Blotting Substrate (Thermo Fisher Scientific), and the densitometry of each band was conducted using ImageJ (National Institutes of Health). The following primary antibodies were used: FABP4 (ab92501, 1:1000, Abcam).

### 2.13 Proteomic analysis

In solution, digestion was used as the sample preparation method as described.^20^ Data acquisition was performed with a Triple‐TOF 6600 mass spectrometer (Sciex, United States) fitted with a Nanospray III source (Sciex, United States). The ion spray voltage was 2300 V, declustering potential 80 V, curtain gas 35 psi, nebulizer gas 5 psi, and interface heater temperature 150°C. Peptides were introduced into the mass spectrometer using Nona 415 liquid chromatography (Sciex, United States). Chromatography solvents were water/acetonitrile/formic acid (A 98/2/0.1%, B 2/98/0.1%). Peptides present in the data were identified by matching to the UniProt Macaca mulatta tcheliensis databases, and corresponding proteins were identified. The Paragon algorithm in Protein Pilot v 5.0 (SCIEX) was used to search the databases. Proteins with a *p*‐value <0.05 or fold change >2 were identified as differentially expressed. Subsequently, GO functional annotation analysis and KEGG pathway analysis of the differentially expressed proteins were performed.

### 2.14 Integrated biological analysis

Gene mRNA and LncRNA expression dataset GSE116925 was downloaded from the Gene Expression Synthesis Library (GEO), a public repository for data storage (http://www.ncbi.nlm.nih.gov/GEO/). We identified the differentially expressed mRNA and LncRNA and performed GO functional annotation analysis and KEGG pathway analysis on the differentially expressed genes. The Venn diagram of differential protein and mRNA was then drawn by the Venn diagram network tool (http://bioinformatics.psb.ugent.be/webtools/Venn/), and the overlapped parts between mRNA and protein were analyzed by Go functional annotation analysis and KEGG pathway analysis. PPI network analysis of overlapped differential genes was then performed using a search tool that retrieved the Interaction Genes (STRING) database. The related lncRNA was also analyzed.

### 2.15 Statistical analysis

All experiments are randomized into groups of similar sample size by block randomization. Data collection and analysis were performed blindly. All the experiments were performed independently at least three times, and data were expressed as mean ± SD. For a comparison of only two groups, we used the Student's *t*‐test. The statistical analyses were conducted by Prism (GraphPad Prism v7.02), and the values of *p* < 0.05 were considered to be statistically significant.

## 3 RESULTS

### 3.1 The proliferation, multilineage differentiation potential, and angiogenic function of BMSCs from different aged mice

We isolated and cultured BMSCs (3 or 4 passages) from 2‐month‐old and 18‐month‐old mice. The expression of MSCs markers, SCA1, CD90, and CD105, were positive in both 2 M BMSCs and 18 M BMSCs (Figure S1A). Meanwhile, the expression of non‐MSCs markers, CD11b, CD45, and CD34, was negative in both 2 M BMSCs and 18 M BMSCs (Figure S1B). Flow cytometric analysis showed that the rate of 18 M BMSCs in bone marrow cells was lower than that of 2 M BMSCs (Figure S2A, B), and the rate of ki67‐positive cells in 18 M BMSCs was lower than that of 2 M BMSCs (Figure S2C, D), indicating that the proliferation rate in vivo of 18 M BMSCs was lower than that of 2 M BMSCs. To investigate the proliferation in vitro of BMSCs of different aged mice, CFSE assays revealed that the proliferation in vitro of 18 M BMSCs was largely declined compared with 2 M BMSCs (Figure S2E, F).

The osteogenic ability of 18 M BMSCs was markedly decreased compared with 2 M BMSCs by Alizarin Red staining and quantitative analysis (Figure S3A, B), and the expression of *ALP* and *Runx*‐*2*, key genes associated with osteogenic differentiation, was significantly downregulated in 18 M BMSCs compared with 2 M BMSCs (Figure S3G, H). The adipogenic ability of 18 M BMSCs was stronger than 2 M BMSCs by Oil Red O staining and quantitative analysis (Figure S3C, D), and the expression of *LPL* and *PPARγ*, key genes associated with adipogenic differentiation, was significantly upregulated in 18 M BMSCs compared with 2 M BMSCs (Figure S3I, J). The chondrogenic ability of 18 M BMSCs was significantly lower than that of 2 M BMSCs by Alcian Blue staining and quantitative analysis (Figure S3E, F). These results proved that the osteogenic and chondrogenic differentiation potential of BMSCs in 18‐month‐old mice is lower than that in 2‐month‐old mice, while the opposite is true for adipogenic differentiation potential.

In addition, we investigated the effects of aging on BMSCs angiogenic potential. After 4h and 8h of incubation, 2 M BMSCs started to interconnect into strips and formed vascular lattice‐like structures, but 18 M BMSCs formed fewer vascular lattice‐like structures (Figure S4A). Meanwhile, the quantitative analysis showed that the tubule length, Nb nodes, and Nb meshes were significantly higher in 2 M BMSCs than in 18 M BMSCs at 4 h and 8 h of incubation (Figure S4B). The expression of *ANG1*, *AQP1*, *CD31*, *HGF*, and *ENG*, key genes associated with angiogenesis, was significantly upregulated in 2 M BMSCs than in 18 M BMSCs (Figure S4C‐G). Taken together, these results indicate that BMSCs in 18‐month‐old mice have weaker angiogenic potential than that in 2‐month‐old mice.

### 3.2 OCR of BMSCs from different aged mice

**FIGURE 1:**
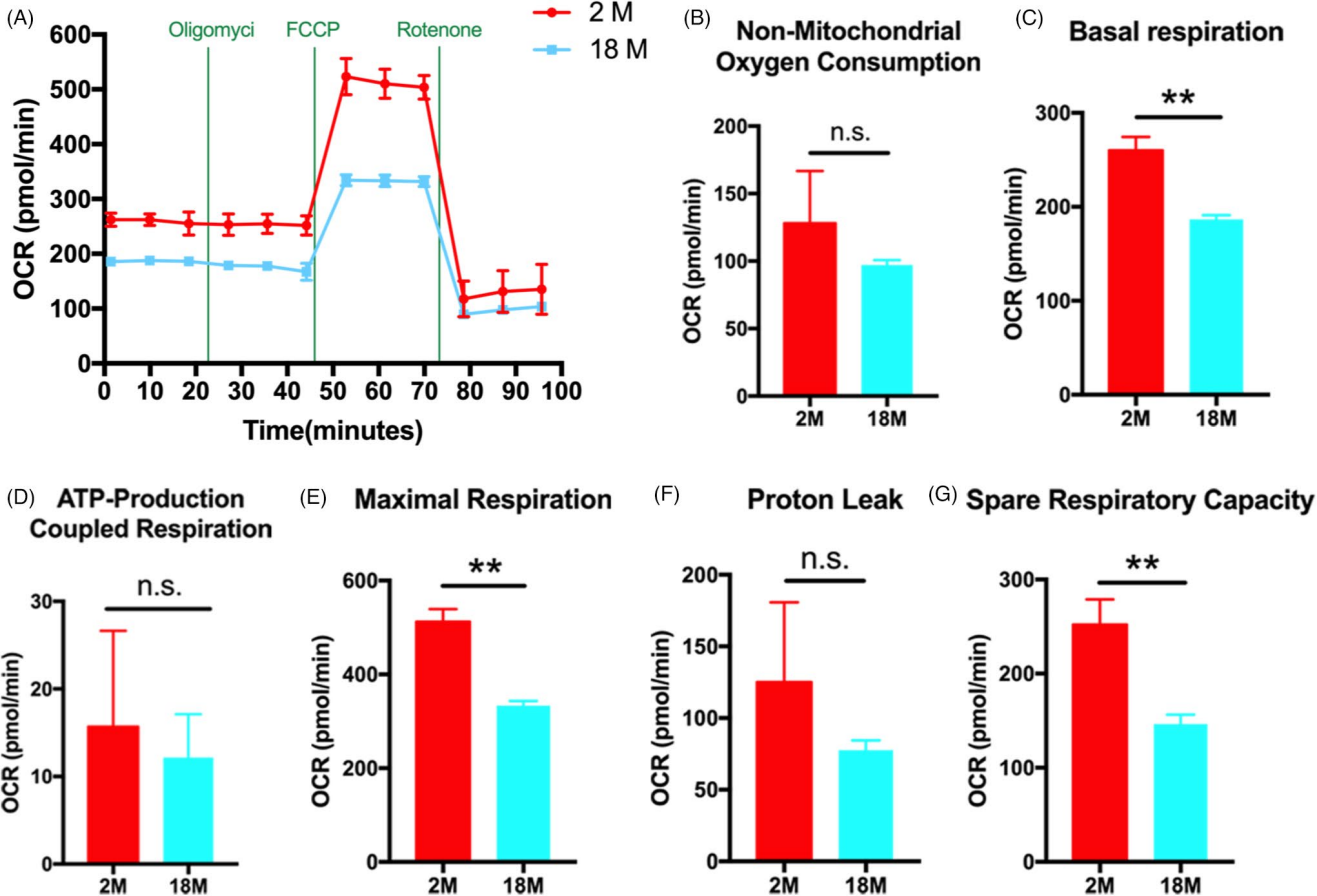
OCR of BMSCs from different aged mice. (A): OCR measurements were obtained. A total of 1‐mM oligomycin, 3‐mM FCCP, and 0.5‐mM antimycin with rotenone were injected sequentially to the sample plate at the time points indicated. (B‐G): OCR values are presented as a bar graph. Data are presented as mean ± SD, *n* = 3 (*p < 0.05, **p < 0.01, n.s., no significance)

To determine the functional changes in cellular metabolic activity of BMSCs, we evaluated the oxygen consumption rate (OCR) of 2 M BMSCs and 18 M BMSCs (Figure 1A), a measure of overall mitochondrial respiration. Our data showed the basal respiration of 18 M BMSCs was significantly lower than that of 2 M BMSCs (Figure 1C). The increase of oxygen consumption after adding uncoupling agent FCCP reflected the mitochondrial reserve function. We found that the maximal respiration of 18 M BMSCs after adding FCCP was significantly lower than that of 2 M BMSCs (Figure 1E). When rotenone was added, the spare respiratory capacity of 18 M BMSCs was significantly lower than that of 2 M BMSCs (Figure 1G). Moreover, compared with 2 M BMSCs, the nonmitochondrial oxygen consumption, ATP production, and proton leakage of 18 M BMSCs were significantly reduced, although there was no significant statistical difference (Figure 1B,D,F). Our results suggest that the oxidative phosphorylation of 18 M BMSCs is defective.

### 3.3 ECAR of BMSCs from different aged mice

**FIGURE 2:**
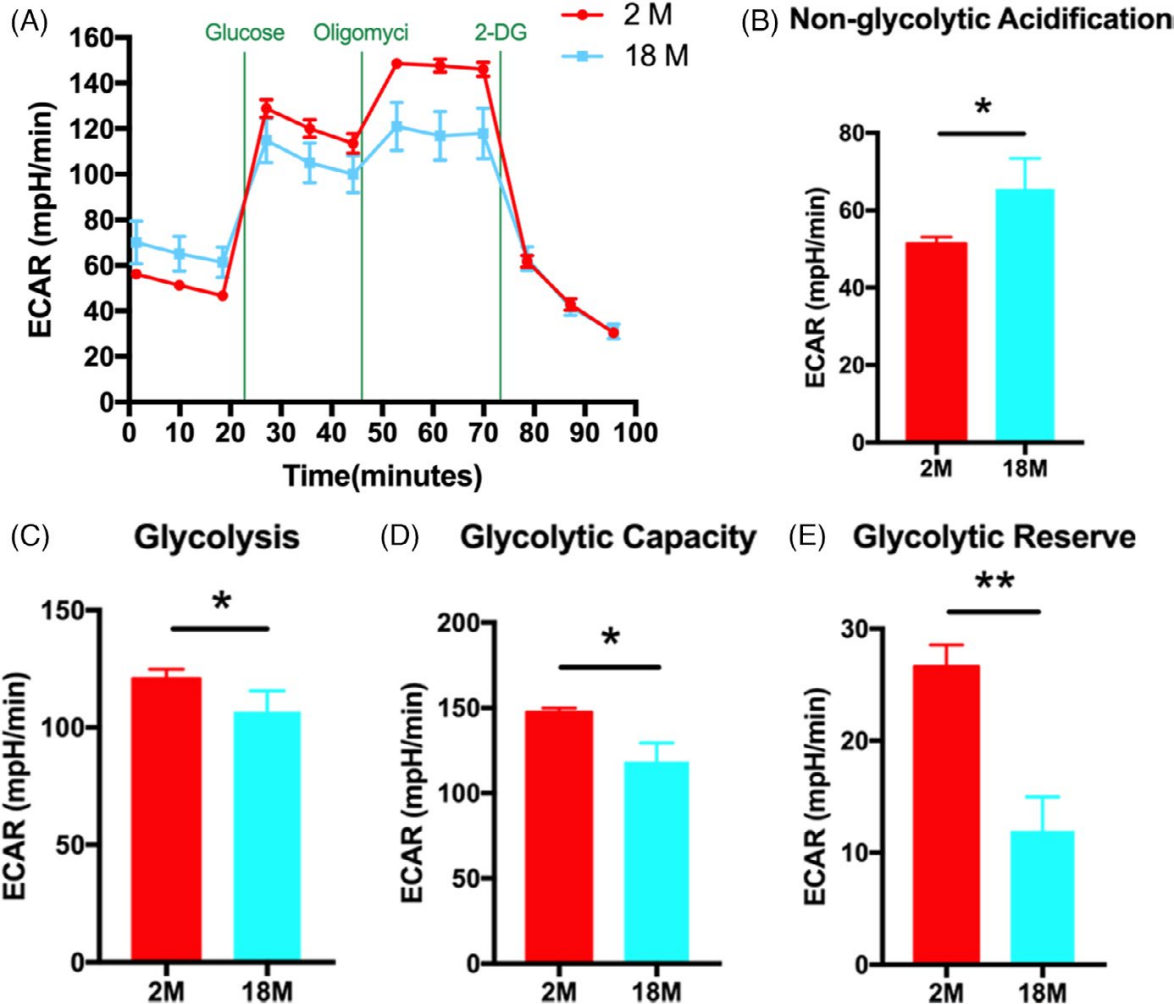
ECAR of BMSCs from different aged mice. (A): ECAR measurements were obtained. A total of 10‐mM glucose, 1‐mM oligomycin, and 50‐mM 2‐DG were injected sequentially to the sample plate at the time points indicated. (B‐E): ECAR values are presented as a bar graph. Data are presented as mean ± SD, *n* = 3 (*p < 0.05, **p < 0.01, n.s., no significance)

To further determine the functional changes in cellular metabolic activity of BMSCs, we evaluated the extracellular acidification rate (ECAR) of 2 M BMSCs and 18 M BMSCs (Figure 2A), which quantifies proton production as a substitute for lactate production. Our data show that the nonglycolytic acidification of 18 M BMSCs is significantly higher than that of 2 M BMSCs (Figure 2B), but the glycolysis of 18 M BMSCs was significantly lower than that of 2 M BMSCs (Figure 2C). When oligomycin was added to stimulate the cells to reach the maximum glycolysis level, the acid production of cells increased, and the increased value represented the glycolysis potential of cells. We found that the glycolytic capacity and glycolytic reserve of 18 M BMSCs were lower than that of 2 M BMSCs (Figure 2D,E). In conclusion, aging may lead to decreasing of metabolic activity pathway in BMSCs.

### 3.4 Gene expression levels of mitochondrial biogenesis and glycolytic key enzymes of BMSCs from different aged mice

**FIGURE 3:**
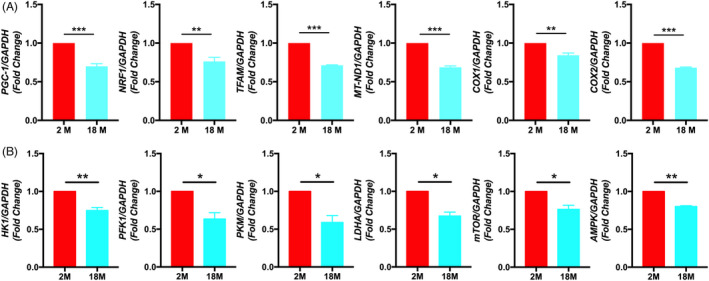
Gene expression levels of mitochondrial biogenesis and glycolytic key enzymes of BMSCs from different aged mice. (A): RT‐PCR analysis showed that compared with 2 M BMSCs, the expression levels of *PGC*‐*1α*, *NRF1*, *TFAM*, *MT*‐*ND1*, *COX1*, *and COX2* in 18 M BMSCs were downregulated. (B): RT‐PCR analysis showed that compared with 2 M BMSCs, the expression levels of *HK1*, *PFK1*, *PKM*, *LDHA*, *mTOR*, and *AMPK* in 18 M BMSCs were downregulated. Data are presented as mean ± SD, *n* = 3 (*p < 0.05, **p < 0.01, n.s., no significance)

In order to further investigate the difference of metabolic activity of BMSCs from different aged mice, we quantitatively analyzed six genes related to mitochondrial biogenesis and 6 genes related to the glycolytic ability of BMSCs, including *PGC*‐*1α*, *NRF1*, *TFAM*, *MT*‐*ND1*, *COX1*, *COX2*, *HK1*, *PFK1*, *PKM*, *LDHA*, *mTOR*, and *AMPK*. The qRT‐PCR results showed that compared with 2 M BMSCs, the expression levels of *PGC*‐*1α*, *NRF1*, *TFAM*, *MT*‐*ND1*, *COX1*, *COX2*, *HK1*, *PFK1*, *PKM*, *LDHA*, *mTOR*, and *AMPK* in 18 M BMSCs were downregulated (Figure 3A, B). Altogether, these results suggest that aging will reduce the expression level of mitochondrial biogenesis and glycolytic key enzyme genes of BMSCs, which may lead to the decrease of BMSCs metabolic activity.

### 3.5 Mitochondria of BMSCs from different aged mice

**FIGURE 4:**
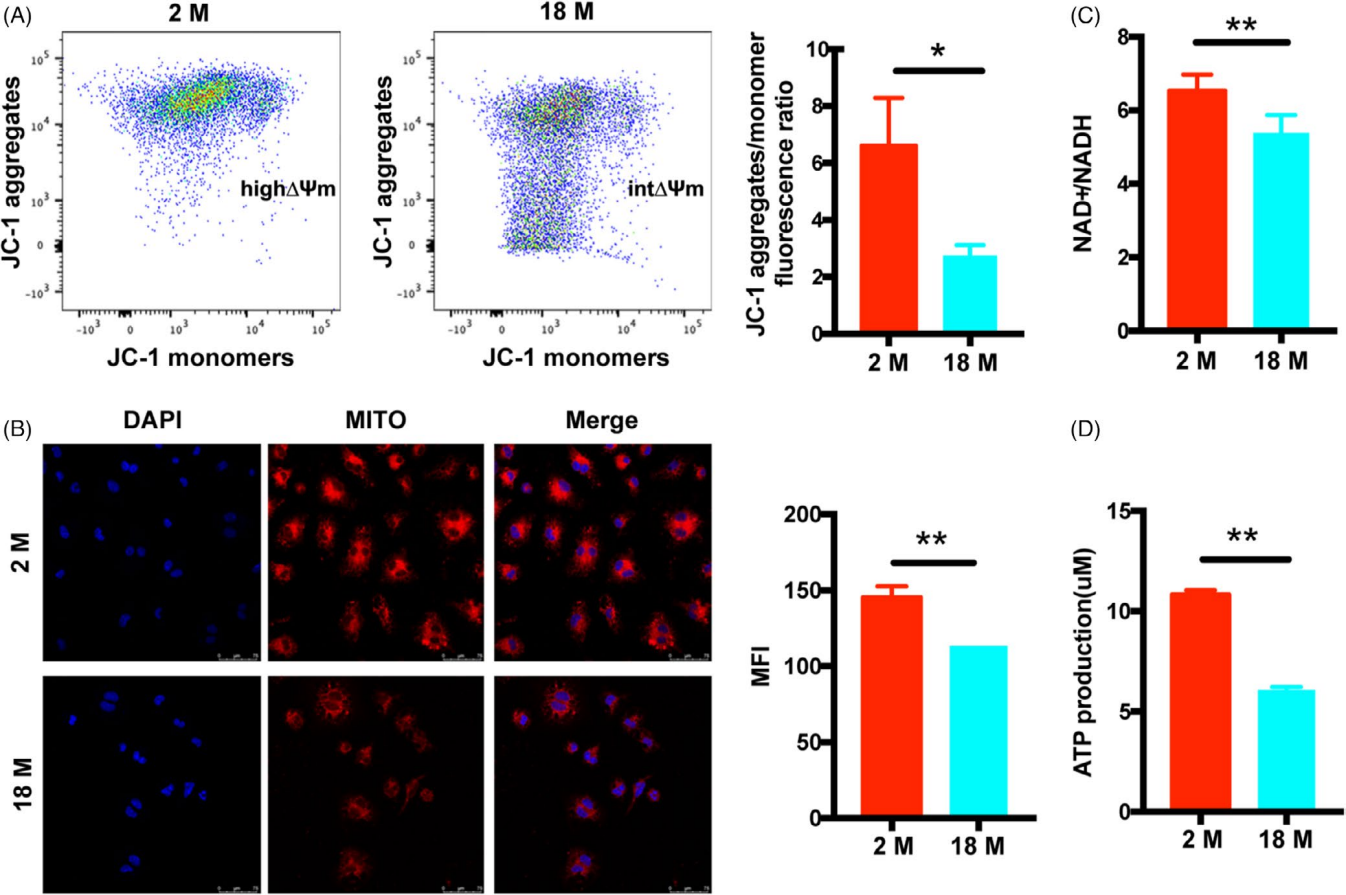
Mitochondrial of BMSCs from different aged mice. (A): The MMP of BMSCs was determined using JC‐1 probes and analyzed. (B): The MMP of BMSCs was determined using MitoTracker Deep Red probes and analyzed. (C): The NAD+/NADH ratio of BMSCs was analyzed. (D): The ATP levels of BMSCs was analyzed. Data are presented as mean ± SD, *n* = 3 (*p < 0.05, **p < 0.01)

As mitochondria are the predominant source for cellular energy, we measured mitochondrial membrane potential (MMP) based on JC‐1 and MitoTracker Deep Red staining. We found that compared with 2 M BMSCs, the MMP was significantly lower in 18 M BMSCs (Figure 4A,B). Moreover, NAD+and NADH maintain a dynamic balance in healthy cells. Our results showed that the NAD+/NADH ratio of 18 M BMSCs was lower than that of 2 M BMSCs (Figure 4C). We further found the significantly reduced ATP levels in 18 M BMSCs compared with those in 2 M BMSCs (Figure 4D). These results collectively support the hypothesis that aging may affect the mitochondrial function of BMSCs.

### 3.6 Integrated biological analysis of BMSCs from different aged mice

**FIGURE 5:**
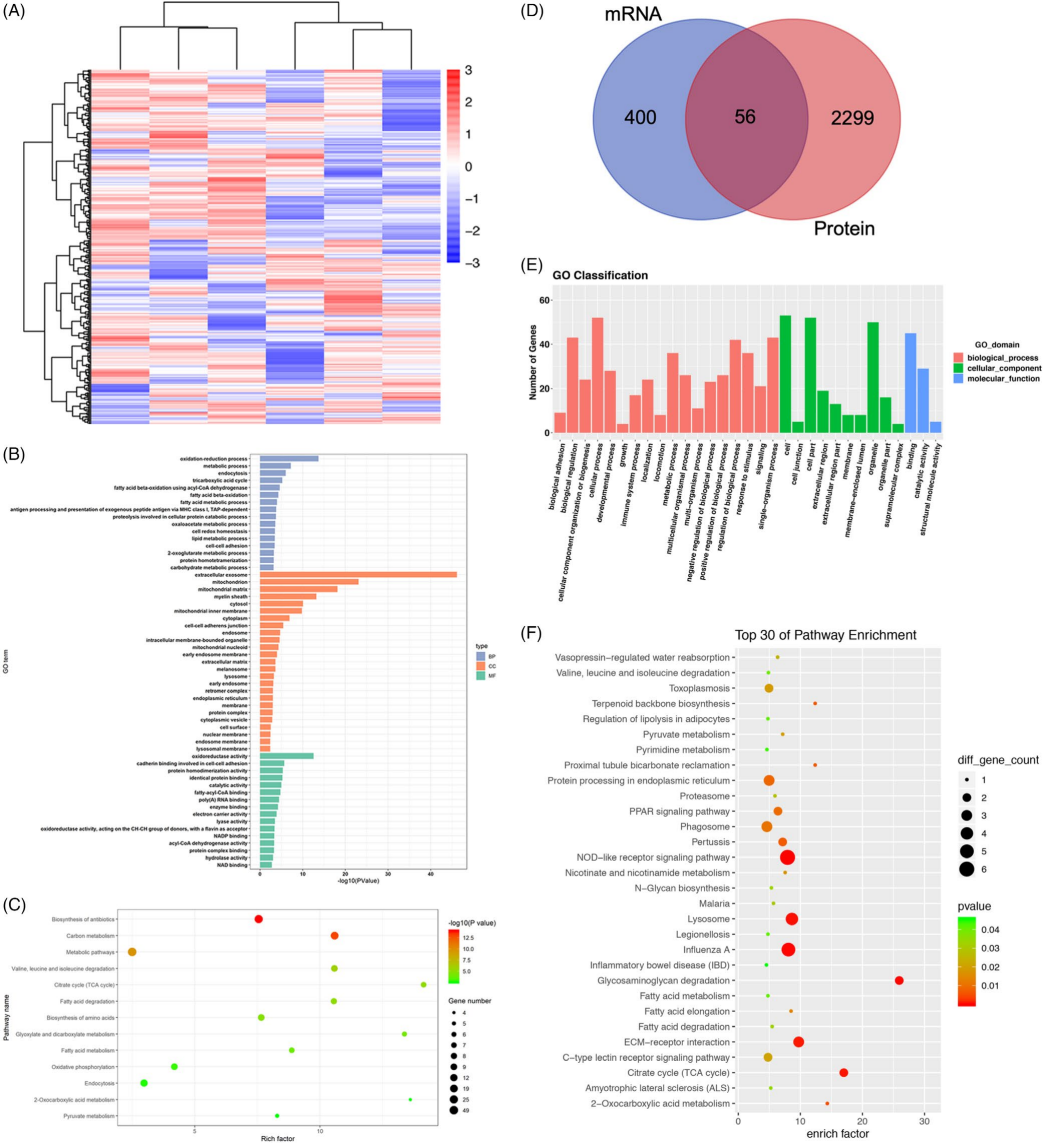
Differentially expressed proteins and mRNA of BMSCs from different aged mice. (A): Cluster analysis of BMSCs in different aged mice revealed differentially expressed proteins. (B): Bar chart showed the number of downregulated proteins based on GO term enrichment. (C): Scatter plot showed the statistical data of downregulated proteins by KEGG pathway enrichment analysis. (D): Venn diagram analysis showed that the overlap and unique different between proteins and mRNA of BMSCs from different aged mice. (E): Bar chart showed the number of overlapped genes based on GO term enrichment. (F): Scatter plot showed the statistical data of overlapped genes by KEGG pathway enrichment analysis

Next, we used proteomic technology to analyze the differentially expressed proteins between 18 M BMSCs and 2 M BMSCs. Our data identify 2358 proteins. In order to identify the precise protein that regulated the mitochondrial function of BMSCs, we combined our dataset with the published mRNA and LncRNA dataset and conducted the integrated analysis (Additional file 6). The differential expressions of proteins, mRNA and LncRNA in 18 M BMSCs and 2 M BMSCs, were analyzed, respectively (Figure 5A and Figure S5A). The differentially expressed proteins, mRNA and LncRNA on GO terms enriched by biological process, cellular component, and molecular function, were showed by the bar chart (Figure 5B and Figure S5B). The differential proteins, mRNA and LncRNA, were then labeled on the KEGG pathway map, and the results of KEGG pathway enrichment analysis were displayed by scatter plot (Figure 5C and Figure S5C). Moreover, we compared the differentially expressed proteins and mRNA by Venn diagram analysis, and 56 proteins and genes were overlapped (Figure 5D). We then analyzed the function of the overlapped 56 proteins/genes by GO terms and KEGG pathway map. The data showed that the differences related to metabolic activity were more significant (Figure 5E,F).

### 3.7 The lipid metabolism function of BMSCs from different aged mice

**FIGURE 6:**
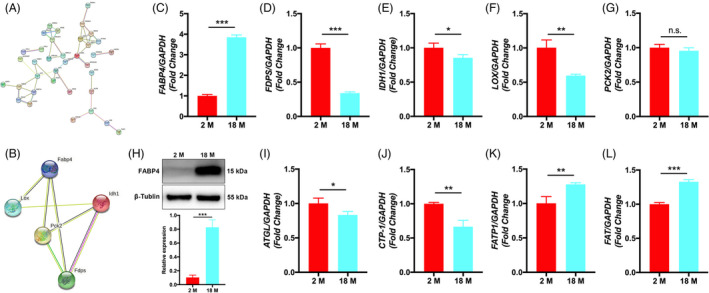
Lipid metabolism function of BMSCs from different aged mice. (A): PPI network map of overlapped differential proteins between proteins and mRNA of BMSCs from different aged mice. (B): PPI network map of five proteins related metabolism function of BMSCs from different aged mice. (C‐G): RT‐PCR analysis showed that compared with 2 M BMSCs, the expression levels of *FABP4* in 18 M BMSCs were upregulated, but *FDPS*, *IDH1*, and *LOX* in 18 M BMSCs were downregulated, *PCK2* was not statistically significant. (H): Western blot analysis showed that the protein levels of FABP4 in 18 M BMSCs was higher than 2 M BMSCs. (I‐L): RT‐PCR analysis showed that compared with 2 M BMSCs, the expression levels of *ATGL* and *CTP*‐*1* in 18 M BMSCs were downregulated, but *FATP1* and *FAT* in 18 M BMSCs were upregulated. Data are presented as mean ± SD, *n* = 3 (*p < 0.05, **p < 0.01, ***p < 0.001, n.s., no significance)

We obtained a PPI network map of the overlapped parts 56 proteins/genes (Figure 6A) and its related LncRNA (Figure S5D). Among them, five proteins, namely FABP4, LOX, PCK2, IDH1, and FDPS, may be the key regulatory protein related to metabolic activity. We then predict their interaction by using the STRING database (Figure 6B). We then analyzed the five proteins and their corresponding LncRNA and found 23 LncRNA co‐expressed with all these five proteins, which may be used as future regulatory targets (Figure S5E). To assess the exact effect of aging on the metabolic activity of BMSCs, we examined the mRNA levels of FABP4, LOX, PCK2, IDH1, and FDPS using qRT‐PCR. We found that the mRNA levels of *FABP4* was upregulated in 18 M BMSCs than in 2 M BMSCs, but the mRNA levels of *FDPS*, *IDH1*, and *LOX* in 18 M BMSCs were all downregulated, in which there was no significant difference in *PCK2* (Figure 6C‐G). Because the most significant difference was found for the mRNA level of *FABP4*, we further examined the changes in its protein levels, and the results showed that the protein levels of FABP4 were significantly higher in 18 M BMSCs than in 2 M BMSCs (Figure 6H). Considering the important role of FABP4 in lipid metabolic process, we concluded that FABP4 might mediate the effects of aging on the metabolic activity of BMSCs. Next, our results showed that 18 M BMSCs downregulated the expression of *ATGL*, a key enzyme of glycolipid metabolism, and inhibited the expression of *CPT*‐*1*, a rate‐limiting enzyme of fatty acid oxidation (Figure 6I, J). Meanwhile, the mRNA levels *FATP1* and *FAT*, fatty acid transport factors, were upregulated in 18 M BMSCs compared with 2 M BMSCs (Figure 6K, L). These results demonstrated that 18 M BMSCs had significantly reduced lipolysis and fatty acid oxidative catabolism compared with 2 M BMSCs.

### 3.8 The function of ROT and CoQ10 on BMSCs from different aged mice

**FIGURE 7:**
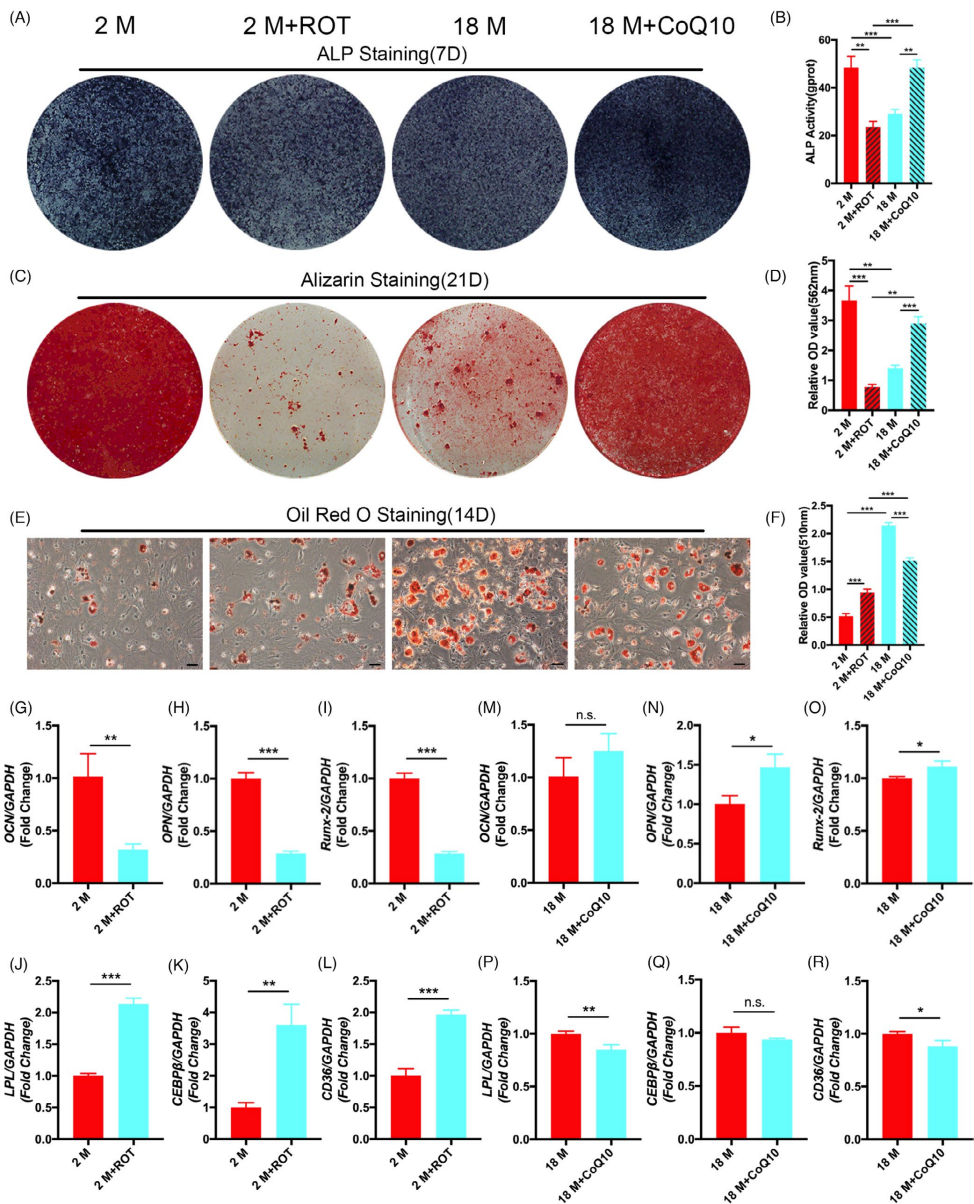
The function of ROT and CoQ10 on BMSCs from different aged mice. (A‐D) Alkaline phosphatase and Alizarin Red staining and quantitative analysis indicated the osteogenic differentiation of ROT inhibition of 2 M BMSCs was declined compared with 2 M BMSCs, whereas the osteogenic differentiation of CoQ10‐activated 18 M BMSCs was stronger than 18 M BMSCs. (E, F) Oil Red O staining and quantitative analysis indicated the adipogenic differentiation of ROT inhibition of 2 M BMSCs was stronger than 2 M BMSCs, whereas CoQ10‐activated 18 M BMSCs was declined compared with 18 M BMSCs. (G‐L) RT‐PCR showed the expression of *OCN*, *OPN*, and *Runx*‐*2* was downregulated in ROT inhibition of 2 M BMSCs compared with 2 M BMSCs, whereas the expression of *LPL*, *CEBPβ*, and *CD36* was upregulated. (M‐R) RT‐PCR showed the expression of *OCN*, *OPN*, and *Runx*‐*2* was upregulated in CoQ10‐activated 18 M BMSCs compared with 18 M BMSCs, whereas the expression of *LPL*, *CEBPβ*, and *CD36* was downregulated. Data are presented as mean ± SD, *n* = 3 (*p < 0.05, **p < 0.01, ***p < 0.001, n.s., no significance)

We blocked or activated the mitochondrial electron transport chain (ETC) of BMSCs using ROT or CoQ10. Alkaline phosphatase and Alizarin Red staining and quantitative analysis showed that the osteogenic differentiation was significantly increased when CoQ10 activated the ETC of 18 M BMSCs; conversely, the osteogenic differentiation was significantly decreased when ROT blocked the ETC of 2 M BMSCs (Figure 7A‐D). The Oil Red O staining and quantitative analysis results indicated that the adipogenic differentiation was significantly reduced when CoQ10 activated the ETC of 18 M BMSCs, whereas the adipogenic differentiation was significantly increased when ROT blocked the ETC of 18 M BMSCs (Figure 7E, F). The changes in the mRNA levels of the cellular osteogenic and adipogenic molecular markers OCN, OPN, Runx‐2, LPL, CEBPβ, and CD36 were consistent with the phenotype (Figure 7G‐R). These results support the metabolic activity may be a potential therapeutic target for enhancing the regenerative functions of BMSCs.

## 4 DISCUSSION

The present study verified that the dysfunction of metabolic activity capacity is positively correlated with the aging process. Our data showed that with increasing age, the proliferation rate, the osteogenic differentiation potential, and immunosuppressive ability of aged BMSCs were decreased. These results agree with the findings of previous studies.^21^, ^22^, ^23^, ^24^ Therefore, our study shows that aging would largely impair the potential of BMSCs for tissue regeneration, which may have unknown mechanisms.

Mitochondria are signal organelles involved in calcium homeostasis, lysosomal transport, inflammation, cell death, and survival signals,^25^, ^26^ as well as biosynthetic organelles of haem, amino acids, and proteins.^27^ The regulation of mitochondria to stem cells is not only about energy conversion, tricarboxylic acid cycle, and oxidative phosphorylation,^28^ but also participate in membrane potential regulation, nucleotide metabolism, and epigenetic markers.^29^ Moreover, protein structural instability and mitochondrial dysfunction can accelerate the aging process.^30^, ^31^

Studies from MSCs have shown that the energy metabolism pattern between mitochondrial oxidative phosphorylation and glycolysis plays an important role in regulating the state of stem cells.^32^ Previous studies have shown that hypoxia or other ways to inhibit mitochondrial oxidative phosphorylation can improve the degree of cell stemness and, on the contrary, by upregulating the key enzymes of the tricarboxylic acid cycle, improving mitochondrial function, or inhibiting glycolysis, can increase ATP production and promote cell differentiation.^33^ Mitochondrial biogenesis is controlled by PGC‐1α, which activates the expression of nuclear respiratory factor (NRF1), further activates mitochondrial transcription factor A (TFAM), and promotes mtDNA replication.^34^ Moreover, NRF1 can also bind to nuclear gene promoter regions, which encode subunits of five complexes (complex I‐V) in mitochondrial electron transport chain (ETC), thereby regulating mtDNA replication.^35^ In addition, glycolytic key enzymes hexokinase (HK), phosphofructokinase (PFK), and pyruvate kinase (PKM) can decompose glucose to produce pyruvate. In anaerobic or hypoxic environment, pyruvate is reduced to lactic acid by lactate dehydrogenase (LDHA) to produce ATP.^36^ Our results showed that the expression levels of mitochondrial biogenesis and glycolytic key enzymes of 18 M BMSCs are reduced, resulting in decreased oxidative phosphorylation and glycolysis, suggesting that the metabolic phenotype of decreased mitochondrial oxidative phosphorylation and glycolysis induced by aging may reduce the stemness and differentiation potential of BMSCs.

Our results also found that the decrease of mitochondrial membrane potential in 18 M BMSCs, which is a landmark event in the early stage of apoptosis, may be related to the decrease of proliferation ability of aging BMSCs. Although there is no simple correlation between membrane potential and superoxide production by the electron transport chain, mitochondria that accumulate in senescence show a decreased membrane potential, suggesting dysfunctionality.^37^ Previous studies have shown that mitochondrial membrane potential is directly related to the age‐related performance of HSCs and that its perturbation has direct consequences for HSC function.^38^ Moreover, the decreased intensity of mitochondrial staining in adipose‐derived mesenchymal stem cells from aged rabbits may be due to reduced mitochondrial activity.^39^ Therefore, our data indicating the mitochondrial membrane potential would be the other target for aged BMSC function restoration.

Recent studies have shown that NAD+, as a coenzyme of redox reaction, is the central molecule of energy metabolism, which can directly or indirectly affect many key cell functions, including metabolic pathways, cell aging, and immune cell function.^40^ Our results showed that NAD+/NADH ratio was decreased in 18 M BMSCs. Since abnormal NAD levels may lead to a variety of diseases, including aging process and inflammatory diseases,^41^, ^42^, ^43^ we hypothesized that aging can weaken the mitochondrial function of BMSCs by reducing the proportion of NAD+. In addition, in eukaryotes, electrons received during glycolysis, oxidative phosphorylation, and oxidation of fatty acids are transferred to the electron transfer chain to form ATP. Disruption of the electron transfer chain (ETC), the core part of mitochondria, results in a decrease in oxidative phosphorylation rate and ATP production. Our results showed that ATP production was damaged in 18 M BMSCs, and OCR results showed the same trend. Our result suggesting that supplement of NAD and promotion of NAD synthesis can regulate mitochondrial dysfunction, which may be a potential target for delaying the aging process of BMSCs.

In order to explore the mechanism leading to the difference, we integrated proteomics, mRNA, and LncRNA biological analysis to identify the genes and related pathways that may play a key role in metabolic activity of BMSCs during the aging process. The overlapped 56 genes/proteins were performed by GO enrichment and KEGG pathway analysis. Our results showed that there were significant differences in metabolic process, pyruvate metabolism, fatty acid metabolism, and citrate cycle (TCA cycle). Among the 56 overlapped genes/proteins, we found that FABP4, LOX, PCK2, IDH1, and FDPS are related to the aging of BMSCs by regulating their metabolic activity. We identified 23 co‐expressed LncRNA with the 5 proteins related to metabolic activity for the construction of lncRNA‐protein co‐expression network. These LncRNA would be the potential regulators of BMSC metabolic activity. Basing on the predict protein and protein interaction, FABP4 would be the key regulator of BMSC mitochondrial function. FABP4 exists widely in tissues and cells, as an important intracellular fatty acid carrier protein participates in the uptake, transportation, esterification, and β‐oxidative capacity.^44^, ^45^ It can also maintain the energy balance of the body, which plays an important role in regulating the normal metabolism of fatty acids and lipid‐mediated signal transduction.^46^ The expression level of ATGL was significantly reduced in 18 M BMSCs compared with 2 M BMSCs. FABP4 could affect the intracellular free fatty acid content, and the reduced free fatty acid concentration could inhibit the expression level of ATGL, a key enzyme of lipolysis, in addition to the increased fatty acid synthesis, which led to the reduction of intracellular lipolysis, while the expression of CPT‐1, the rate‐limiting enzyme of fatty acid mobilization and oxidation, was also significantly reduced in 18 M BMSCs. Moreover, the high expression of FABP4 in 18 M BMSCs can therefore increase the aggregation of fatty acids near the cell membrane, and then, more free fatty acids need to enter or exit the cell, which makes the expression of FATP1 and FAT, two important fatty acid transporters on the cell membrane, increase, thus meeting the need of fatty acid transport. Studies have shown that FABP4‐deficient mice have protective effects on dyslipidemia, hyperglycemia, and insulin resistance,^47^ and interference with FABP4 can promote mitochondrial maintenance of high membrane potential, which is conducive to strengthening mitochondrial function.^48^ Therefore, we speculate that FABP4 may play a role in cell senescence caused by fatty acid metabolism disorders.

Our comprehensive experimental study has confirmed the mitochondrial dysfunction play an important role in BMSCs senescence, which may be an important way to maintain cell activity and function. The identified potential regulatory protein would provide a target for enhancing MSCs function and the efficiency of regenerative medicine.

## 5 CONCLUSIONS

Although the application of MSCs in preclinical and clinical research has provided encouraging results for the treatment of various degenerative diseases, the senescence of MSCs is still a major challenge, which limits the clinical application of MSCs. Our results provide novel insights into the differences in the metabolism characteristics of BMSCs from young and elderly mice and elucidate the potential mechanism of mitochondrial dysfunction‐related aging. Our results would contribute to understand these mechanisms of senescence of MSCs and enhance MSC‐based tissue engineering and regenerative medicine.

## CONFLICT OF INTEREST

The authors have declared that no competing interest exists.

## AUTHOR CONTRIBUTIONS

SW and LH conceived and designed the experiments. XL and LH performed the experiments. XW and LH assisted the experiments. XL and LH analyzed the data. XL wrote the paper. SW and LH revised the manuscript. All authors read and approved the manuscript.

## Supporting information

Fig S1

Fig S2

Fig S3

Fig S4

Fig S5

Table S1

Supplementary Material

## Data Availability

All data and materials associated with this study are present in this published article.
