# Supplementary material for: Dysfunction of metabolic activity of bone marrow mesenchymal stem cells in aged mice
Source: Cell Prolif. 2022 Jan 27;55(3):e13191. doi: 10.1111/cpr.13191 (PMC8891618; doi:10.1111/cpr.13191)
Supplement: Supplementary file 1 — Fig S1 [file CPR-55-e13191-s005.docx]

**Additional file 2**

**Fig. S1.**

**
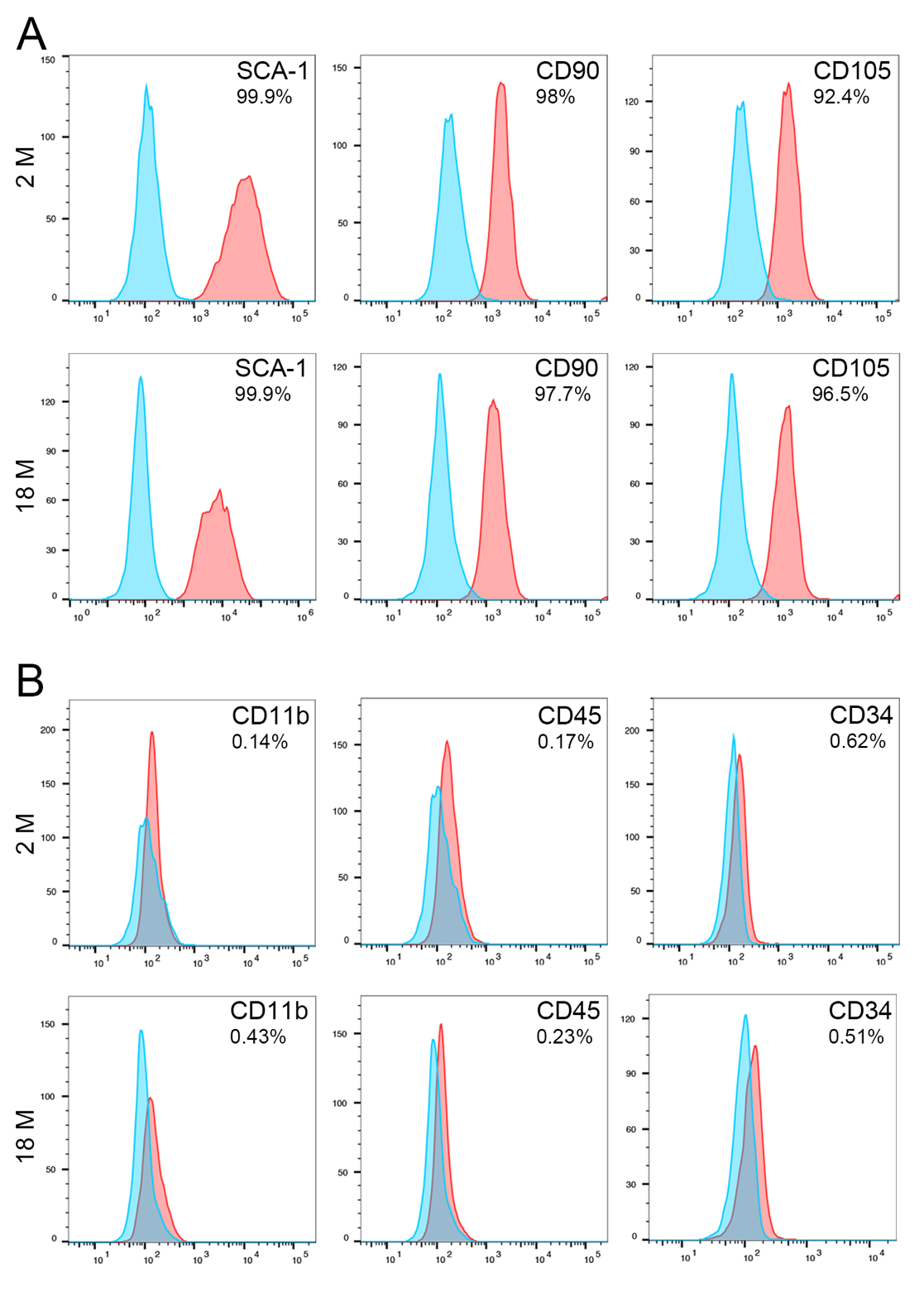
**

**Fig. S1. Characterization of BMSCs from different age mice**

**(A) Both 2M BMSCs and 18M BMSCs expressed SCA1, CD90 and CD105 positively. (B) Both 2M BMSCs and 18M BMSCs expressed CD11b, CD45 and CD34 negatively.**
