# Supplementary material for: Dysfunction of metabolic activity of bone marrow mesenchymal stem cells in aged mice
Source: Cell Prolif. 2022 Jan 27;55(3):e13191. doi: 10.1111/cpr.13191 (PMC8891618; doi:10.1111/cpr.13191)
Supplement: Supplementary file 2 — Fig S2 [file CPR-55-e13191-s002.docx]

**Additional file 3**

**Fig. S2.**

**
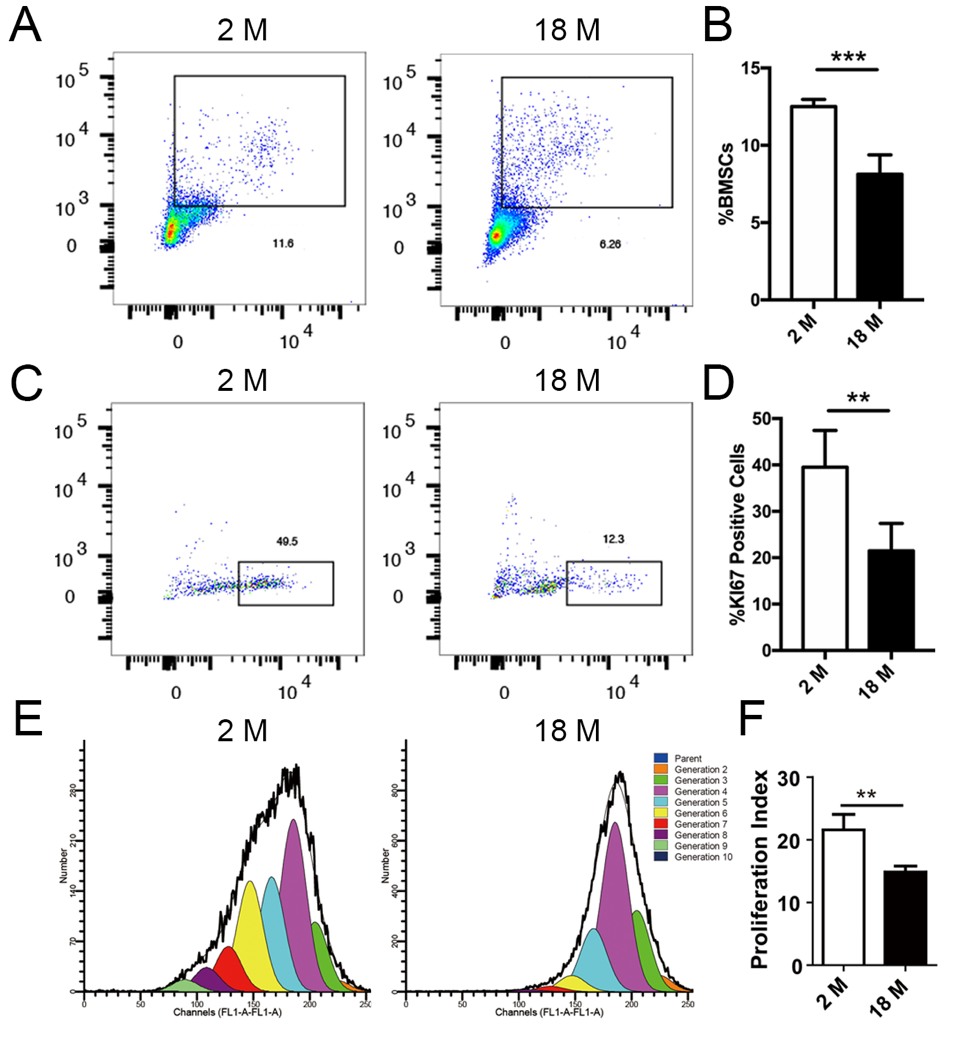
**

**Fig. S2. Proliferation of BMSCs from different aged mice**

**(A, B) The proportion of 18M BMSCs in bone marrow cells was lower than that of 2M BMSCs. (C, D) Ki67 staining indicated 18M BMSCs had a lower proliferation rate in vivo than 2M BMSCs. (E, F) CFSE assay showed the proliferation in vitro of 18M BMSCs was declined compared with 2M BMSCs. Data are presented as mean ± SD, n=3 (**P < 0.01).**
