# Supplementary material for: Dysfunction of metabolic activity of bone marrow mesenchymal stem cells in aged mice
Source: Cell Prolif. 2022 Jan 27;55(3):e13191. doi: 10.1111/cpr.13191 (PMC8891618; doi:10.1111/cpr.13191)
Supplement: Supplementary file 3 — Fig S3 [file CPR-55-e13191-s007.docx]

**Additional file 4**

**Fig. S3**

**
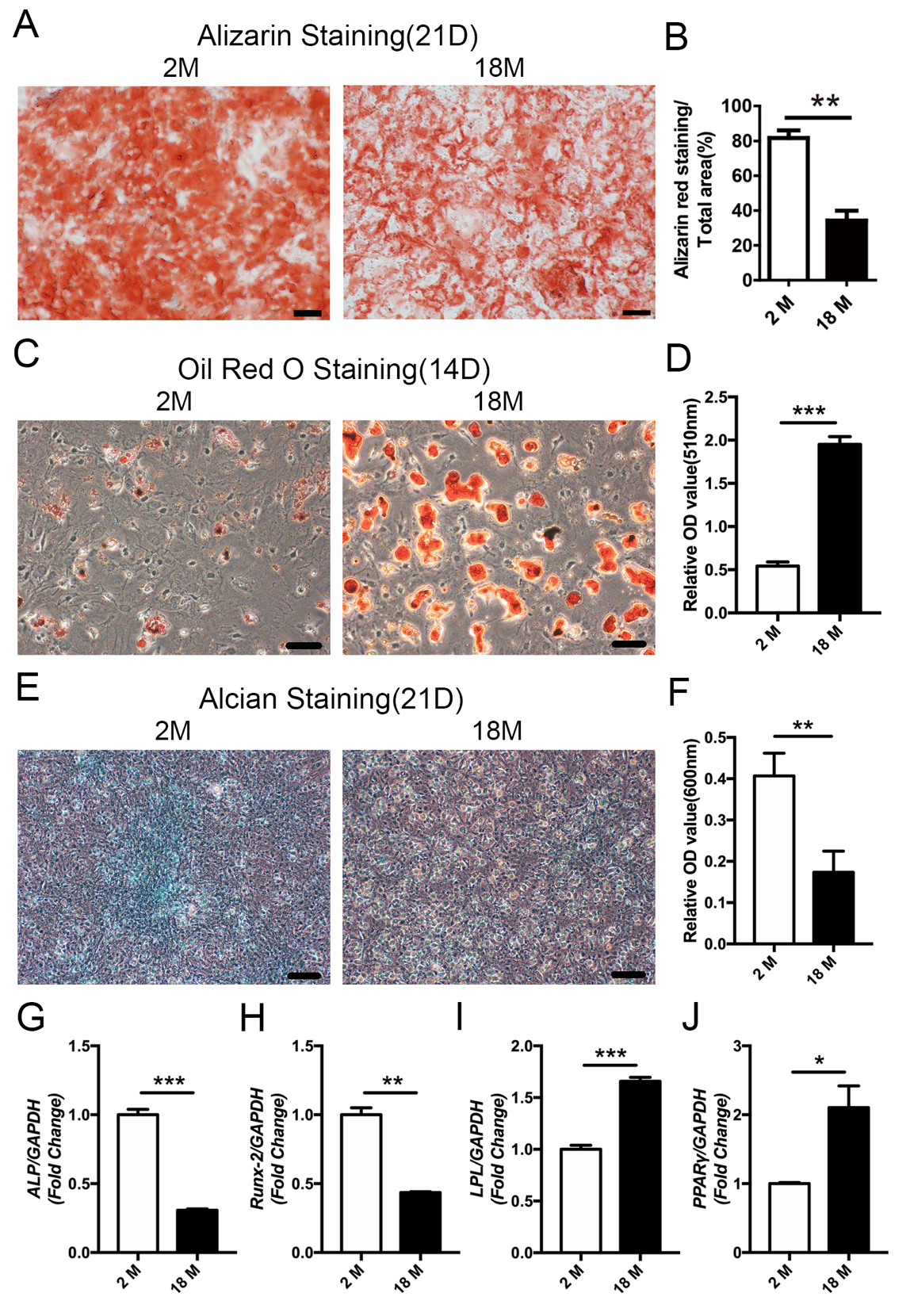
**

**Fig. S3 Multilineage differentiation of BMSCs from different aged mice**

**(A, B) Alizarin red staining and quantitative analysis showed the osteogenic differentiation of 18M BMSCs was declined compared with the 2M BMSCs. (C, D) Oil Red O staining and quantitative analysis showed the adipogenic differentiation of 18M BMSCs was stronger than 2M BMSCs. (E, F) Alcian Blue staining and quantitative analysis showed the chondrogenic differentiation of 18M BMSCs was lower than that of 2M BMSCs. (G, H) RT-PCR showed the expression of *ALP* and *Runx-2* was down-regulated in 18M BMSCs compared with 2M BMSCs. (I, J) RT-PCR showed the expression of *LPL* and *PPARγ* was upregulated in 18M BMSCs compared with 2M BMSCs. Data are presented as mean ± SD, n=3 (*P < 0.05, **P < 0.01, ***P < 0.001).**
