# Supplementary material for: Dysfunction of metabolic activity of bone marrow mesenchymal stem cells in aged mice
Source: Cell Prolif. 2022 Jan 27;55(3):e13191. doi: 10.1111/cpr.13191 (PMC8891618; doi:10.1111/cpr.13191)
Supplement: Supplementary file 4 — Fig S4 [file CPR-55-e13191-s004.docx]

**Additional file 5**

**Fig. S4**

**
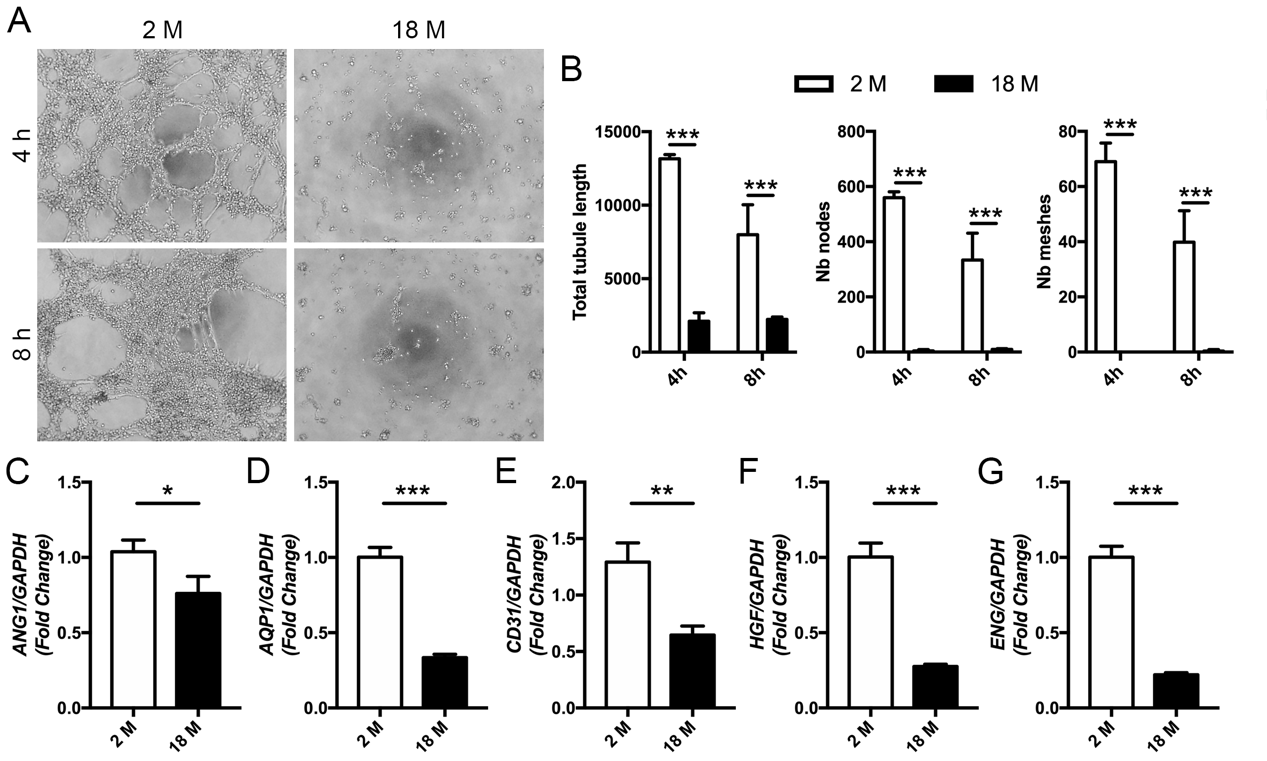
**

**Fig. S4 Angiogenic potential of BMSCs from different aged mice**

**(A, B) 2M BMSCs formed more vascular lattice-like structures than 18M BMSCs. (C-G) The expression level of *ANG1*, *AQP1*, *CD31*, *HGF*, and *ENG* was declined in 18M BMSCs compared with 2M BMSCs. Data are presented as mean ± SD, n=3 (*P < 0.05, **P < 0.01).**
