# Supplementary material for: Dysfunction of metabolic activity of bone marrow mesenchymal stem cells in aged mice
Source: Cell Prolif. 2022 Jan 27;55(3):e13191. doi: 10.1111/cpr.13191 (PMC8891618; doi:10.1111/cpr.13191)
Supplement: Supplementary file 5 — Fig S5 [file CPR-55-e13191-s001.docx]

**Additional file 7**

**Fig. S5**


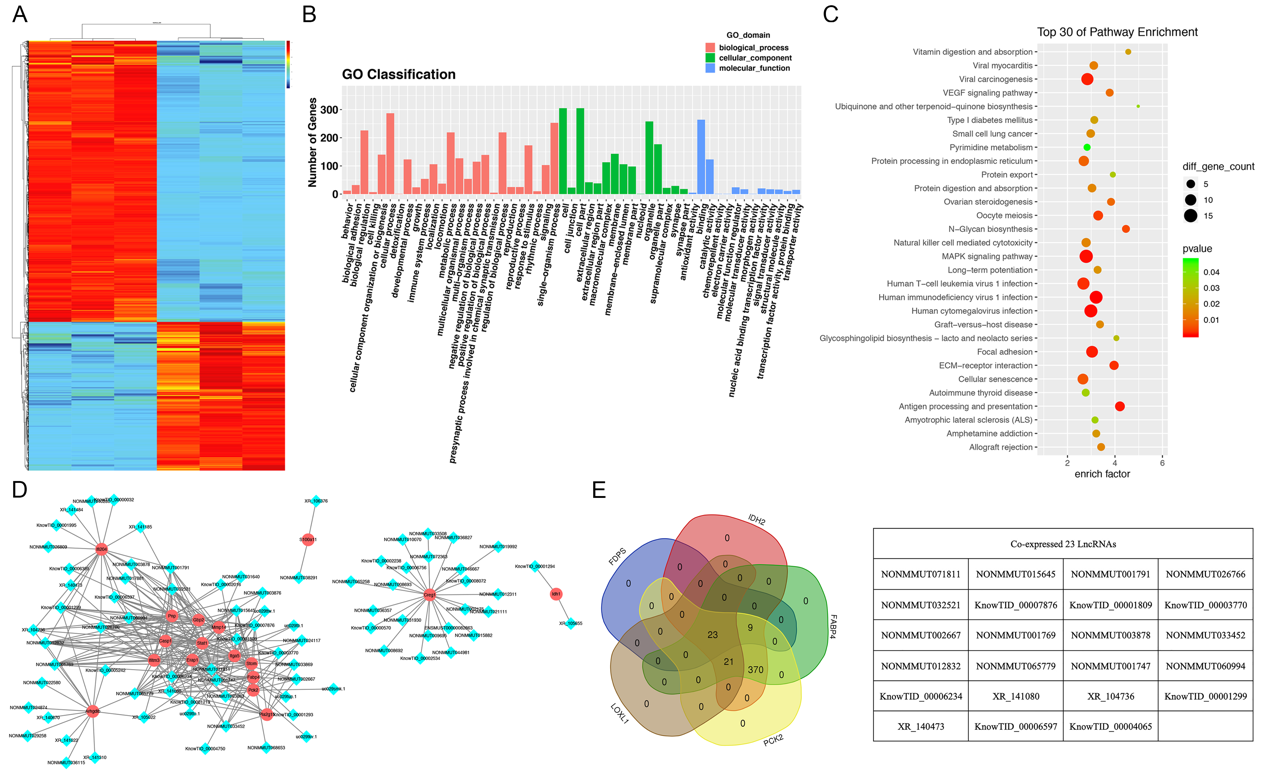


**Fig. S5 Differentially expressed mRNA and LncRNA of BMSCs from different aged mice.**

**(A) Cluster analysis of BMSCs in different aged mice revealed differentially expressed mRNA and LncRNA.**

**(B) Bar chart showed the number of mRNA and LncRNA based on GO term enrichment.**

**(C) Scatter plot showed the statistical data of mRNA and LncRNA by KEGG pathway enrichment analysis.**

**(D): PPI network map of LncRNA and proteins of BMSCs from different aged mice.**

**(E): Venn diagram analysis showed that the overlap and unique different between five proteins and LncRNA of BMSCs from different aged mice.**
