# Supplementary material for: Dysfunction of metabolic activity of bone marrow mesenchymal stem cells in aged mice
Source: Cell Prolif. 2022 Jan 27;55(3):e13191. doi: 10.1111/cpr.13191 (PMC8891618; doi:10.1111/cpr.13191)
Supplement: Supplementary file 6 — Table S1 [file CPR-55-e13191-s003.docx]

**Additional file 1**

**Table 1** Gene primers

| Gene names | Sense primers | Antisense primers |
| --- | --- | --- |
| ALP  OCN  OPN  Runx-2  LPL  CEBPβ  CD36  ANG1  AQP1  CD31  HGF  ENG  PGC-1α  NRF1  TFAM  MT-ND1  COX1  COX2  HK1 | CCAACTCTTTTGTGCCAGAGA  AATCCGGACTGTGACGAGTTG  AAGCATCCTTGCTTGGGTTTG  TCGGAGAGGTACCAGATGGG  AGATGCCCTACAAAGTGTTCCA  CGCCTTATAAACCTCCCGCT  GGAGCAACTGGTGGATGGTT  CAGTGGCTGCAAAAACTTGA  CCGAGACTTAGGTGGCTCAG  AGAGCCAGCATTGTGACCAGTC  CGAGCTATCGCGGTAAAGAC  TGCAGAAAGAGTCGGTTGTG  GTCATGTGACTGGGGACTGT  TGGCTGATGGAGAGGTGGAA  GTATTGCGTGAGACGAACCG  CTCAACCTAGCAGAAACAAACC  TCAGTATCGTATGCTTCAACAAATTTAGA  GAGCAGTCCCCTCCCTAGGA  CAGCTCCTGGCCTATTACTTCA | GGCTACATTGGTGTTGAGCTTTT  CAGCAGAGCGACACCCTAGAC  ATGGTCGTAGTTAGTCCCTCAGA  AGGTGAAACTCTTGCCTCGT  ATTTGTGGAAACCTCGGGCA  TGGCCACTTCCATGGGTCTA  CTACGTGGCCCGGTTCTAAT  TCCACATCTGTGAGCTTTCG  TCATGCGGTCTGTAAAGTCG  CAAGGCGGCAATGACCACTCC  TGTAGCTTTCACCGTTGCAG  TCTCAGTGCCATTTTGCTTG  AACCAGAGCAGCACACTCTAT  TTCTGCCAGTGATGCTACCG  CTTCGGAATACAGACAAGACTGA  GGCCGGCTGCGTATTCTAC  TGGTTCCTCGAATGTGTGATATG  GTCGGTTTGATGTTACTGTTGCTT  GAAATCTCCCTTTTCAGAGCCA |
| PFK1 | CAGAAACCCAGTACACCCCC | AACTGGGGTATGGATGAGCC |
| PKM | TTCGTCTTTGCAGCGTAGCC | TCCCTTCTTCAGCTCCACTGAT |
| LDHA | CTGGCAAAGTGGATATCTTGAC | ACTCCATACAGGCACACTGG |
| HIF-1α | TTTGGCAGCAACGACACAGA | CGTTTCAGCGGTGGGTAATG |
| GLUT1 | TCTGGCATCAACGCTGTCTTC | AAACAGCGACACGACAGTGA |
| MYC | CCCCTACCCTCTCAACGACA | CTTCTTGTTCCTCCTCAGAGTCG |
| mTOR | GACCTCTTCTCCTTGGCACA | CTTCTGGTGTCAGGGTATCCCATA |
| AMPK  FABP4  FDPS  IDH1  LOX  PCK2  ATGL  CTP-1  FATP1  FAT | TCGGCAAAGTGAAGGTTGGC  ACGACAGGAAGGTGAAGAGCAT  GCGTTGAAGAACAGGGAGTG  GTAGTCCAGAGTGAAGAGGGTT  GTAACTGCAAACTGCCACGTC  ATGGTGGTAACTCCTTGCTGG  CCTTAGGAGGAATGCCCTGC  GACTCCGCTCGCTCATTCC  GGATCAGGACTAGGTGGGAG  GGAGCAACTGGTGGATGGTT | CAGATGGTGTACTGATGACCTGG  AACTCTTGTGGAAGTCACGCC  GGGAGGGTCGTTGTAGGAAA  TGGTGGCATCACGATTCTCT  CTGCCCGTTGTTCTCCCATT  ATGGCCCGGAGTTGACCTTC  CTCCAGCGGCAGAGTATAGG  ACCAGTGATGATGCCATTCTTG  GCCGAACACGAATCAGAACAG  CTACGTGGCCCGGTTCTAAT |
| GAPDH | TGACCTCAACTACATGGTCTACA | CTTCCCATTCTCGGCCTTG |

ALP: alkaline phosphatase; OCN: osteocalcin; OPN: osteopontin; Runx-2: runt-related transcription factor 2; LPL: lipoprotein lipase; CEBPβ: CCAAT/enhancer binding protein β; CD36: cluster of differentiation 36; ANG1: angiopoietin-1; AQP1: aquaporin 1; CD31: platelet/endothelial cell adhesion molecule 31; HGF: hepatocyte growth factor; ENG: endoglin; PGC-1α: peroxlsome proliferator-activated receptor-γ coactlvator-1α; NRF1: nuclear respiratory factor 1; TFAM: mitochondrial transcription factor A; MT-ND1: mitochondrially encoded NADH dehydrogenase subunit 1; COX1: cytochrome c oxidase subunit 1; COX2: cytochrome c oxidase subunit 2; HK1: hexokinase 1; PFK1: 6-phosphofructokinase1; PKM: pyruvate kinase M; LDHA: lactate dehydrogenase A; mTOR: mammalian target of rapamycin; AMPK: adenine monophosphate activated protein kinase; FABP4: fatty acid-binding protein 4; FDPS: farnesyl diphosphate synthase; IDH1: isocitrate dehydrogenase (NADP(+)) 1; LOX: lysyl oxidase; PCK2: phosphoenolpyruvate carboxykinase 2; ATGL: atglistatin; CTP-1: cytidine triphosphate 1; FATP1: fatty acid transport protein 1; FAT: fatty acid transport; GAPDH: glyceraldehyde 3-phosphate dehydrogenase.
